# Combined Effects of Temperature and Toxic Algal Abundance on Paralytic Shellfish Toxic Accumulation, Tissue Distribution and Elimination Dynamics in Mussels *Mytilus coruscus*

**DOI:** 10.3390/toxins13060425

**Published:** 2021-06-17

**Authors:** Yunyu Tang, Haiyan Zhang, Yu Wang, Chengqi Fan, Xiaosheng Shen

**Affiliations:** East China Sea Fisheries Research Institute, Chinese Academy of Fishery Sciences, Jungong 300, Shanghai 200090, China; tangyunyu@ecsf.ac.cn (Y.T.); zhanghaiyan@ecsf.ac.cn (H.Z.); wangy0513@126.com (Y.W.); fancq@ecsf.ac.cn (C.F.)

**Keywords:** harmful algal blooms, paralytic shellfish toxins, pharmacokinetics, environmental changes, *A. catenella*

## Abstract

This study assessed the impact of increasing seawater surface temperature (SST) and toxic algal abundance (TAA) on the accumulation, tissue distribution and elimination dynamics of paralytic shellfish toxins (PSTs) in mussels. *Mytilus coruscus* were fed with the PSTs-producing dinoflagellate *A. catenella* under four simulated environment conditions. The maximum PSTs concentration was determined to be 3548 µg STX eq.kg^−1^, which was four times higher than the EU regulatory limit. The increasing SST caused a significant decline in PSTs levels in mussels with rapid elimination rates, whereas high TAA increased the PSTs concentration. As a result, the PSTs toxicity levels decreased under the combined condition. Additionally, toxin burdens were assessed within shellfish tissues, with the highest levels quantified in the hepatopancreas. It is noteworthy that the toxin burden shifted towards the mantle from gill, muscle and gonad at the 17th day. Moreover, variability of PSTs was measured, and was associated with changes in each environmental factor. Hence, this study primarily illustrates the combined effects of SST and TAA on PSTs toxicity, showing that increasing environmental temperature is of benefit to lower PSTs toxicity with rapid elimination rates.

## 1. Introduction

Harmful algal blooms (HABs) are growing into global environmental problems and have negative impacts on marine resources and human health [1,2,3,4]. Paralytic shellfish poisoning (PSP) is generated through the ingestion of toxic shellfish contaminated by paralytic shellfish toxins (PSTs), resulting in people suffering from illness and death [5,6,7,8]. Currently, gastric lavage is regarded as the only therapy to treat the poisoning patients. On a global scale, there are about 2000 PSP incidence each year, accounting for 15% fatalities [9]. Moreover, besides the high ecological importance of bivalves, they are also important protein sources for human consumption, playing a key role in life. Thus, PSTs are detrimental to economies of the aquaculture industry and human health.

In the past, numerous investigations on the accumulation and elimination of PSTs within mussels [10,11], scallops [12,13], clams [14,15,16], and oysters [17,18] have been reported. The greatest variations were observed in various shellfish, with the toxin duration time lasting from days to months [19,20,21,22]. Among them, mussels are known to accumulate high levels of toxins faster than most other species, as well as having faster elimination rates [23,24,25,26]. Additionally, the burden or amount of toxicity varies in different organs based on the toxin exposure time. Generally, the viscera of bivalves, including the hepatopancreas, are the major repository of PSTs, showing the highest toxicity of about 80−98% [27,28,29]. As for the depuration, the elimination rates are closely related to the different analogues of PSTs to a large extent [30,31,32].

It is noteworthy that increasing seawater surface temperature (SST) caused by environmental change is estimated to get worse by the end of this century, with it rising 5 °C in some areas [33,34]. Meanwhile, changes of behavior, immune response, metabolic rate and reduction of growth rates were observed in mussels [35,36]. On the other hand, high temperature is beneficial for toxic algae growth [37], significantly affecting the accumulation and elimination of PSTs in bivalves [38,39,40]. Along the Chinese coast, in Guangdong and Fujian provinces, as well as the northern part of the Yellow Sea, shellfish were more likely to be contaminated by PSTs [41,42,43]. It has been found that toxicity levels of shellfish are mainly affected by the toxic algae species, abundance, and exposure time. Generally, species belonging to the dinoflagellate *Alexandrium*, *Gymnodinium* and *Pyrodinium* genera are reported to produce PSTs [44,45,46,47,48,49]. Among them, genus *Alexandrium* harbors the majority of the producers. In this respect, *Alexandrium tamarense* (ATHK) is widely distributed along the Chinese coasts from the Bohai Sea to the South China Sea, causing the blooms of toxic *Alexandrium* spp. [50,51]. However, limited information is available on the impacts for shellfish toxicity, and study of assessing effects under different abundances of toxic algae has not been investigated, which are important for understanding the mechanisms of shellfish toxicity accumulation and maintaining seafood safety.

**Figure 1:**
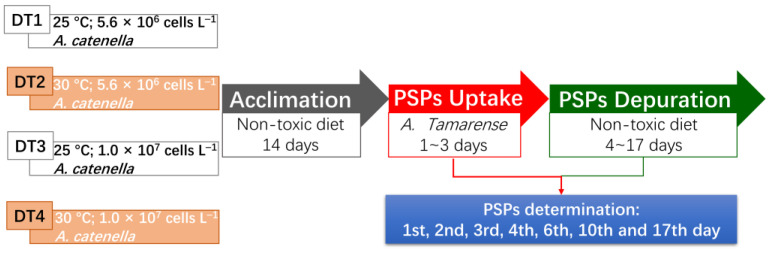
Schematic diagram of the experiment set-ups.

This study aims to assess the effect of the increasing seawater surface temperature and *A. catenella* (strain ATHK) abundance on the dynamics of PSTs accumulation and elimination in *Mytilus coruscus* (Figure 1).

## 2. Results

### 2.1. Toxin of A. catenella

The results from the LC-MS/MS for the PSTs analogues were found to be GTX1+4, C1+2, GTX5, NEO, dcGTX2+3, GTX2+3, STX and dcSTX in different concentrations produced by *A. catenella* (Table 1). GTX1+4 (51.6%) was the most abundant toxin, followed by C1+2 (38.9%), GTX5 (6.7%) and NEO (1.9%). Moreover, the strain also produced the dcGTX2+3 (0.4%) and GTX2+3 (0.3%). Additionally, dcSTX and STX were detected with the percentage accounting for only 0.1%.

### 2.2. Effect of Temperature Increase and Toxic Algal Abundance on Mussels

**Figure 2:**
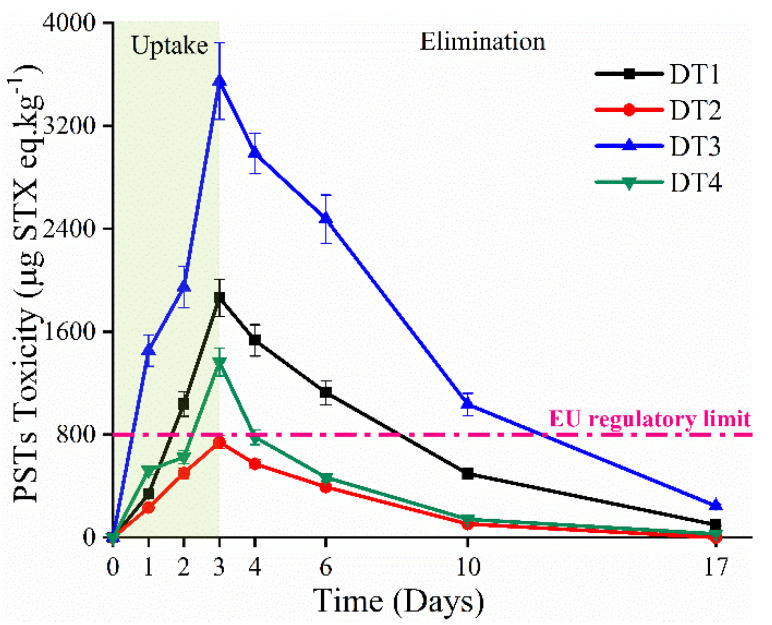
PSTs (µg STX eq. kg^−1^, mean ± SD) detected in mussels during the experiment under four environmental conditions (DT1: 25 °C and C*_A. catenella_* 5.6 × 10^6^ cells L^−1^; DT2: 30 °C and C*_A. catenella_* 5.6 × 10^6^ cells L^−1^; DT3: 25 °C and C*_A. catenella_* 1.0 × 10^7^ cells L^−1^; DT4: 30 °C and C*_A. catenella_* 1.0 × 10^7^ cells L^−1^).

Obvious changes in PSTs accumulation were observed in mussels under four conditions (Figure 2). The highest toxin level was detected under a high TAA condition (DT3: 25 °C and 1.0 × 10^7^ cells L^−1^ *A. catenella*, Figure 1), reaching levels of 3548 ± 299 μg STX eq. kg^−1^, expressed as saxitoxin equivalents after multiplying the toxin concentration with the respective toxicity factor (Table S2). After that, a level of 1864 ± 144 μg STX eq. kg^−1^ was obtained under the basic condition (DT1: 25 °C and 5.6 × 10^6^ cells L^−1^ *A. catenella*). PSTs toxicity level under high SST condition (DT2: 30 °C and 5.6 × 10^6^ cells L^−1^ *A. catenella*, 741 ± 51 μg STX eq. kg^−1^) showed a lower value compared to that of DT1. However, the combined effect of high SST and TAA (DT4: 30 °C and 1.0 × 10^7^ cells L^−1^ *A. catenella*), resulted in a much higher PSTs value (1363 ± 108 μg STX eq. kg^−1^) than that of DT2. 

On the other hand, the PSTs toxicities were determined in various tissues of mussels, such as hepatopancreas, gill, mantle, muscle and gonad throughout the experiment. Similar toxicity tendency under four conditions was observed for in all tissues (Figure S1). After exposure to toxic algae for 3 days, the highest and lowest toxicity levels were observed in DT3 and DT2 for all tissues, respectively. The toxicity levels rank in the order of DT2 < DT4 < DT1 < DT3 in the hepatopancreas and mantle, which are in accordance with the order of the total toxicity levels (Figure 2). However, some different observations were obtained in gill, gonad and muscle, exhibiting nearly the same toxicity values in DT1 and DT4, even higher levels in DT4 than that of DT1 in muscle. As expected, most PSTs were concentrated in the hepatopancreas, followed by gill and mantle. The gonad and muscle showed similar toxicity values, which are significantly lower than that of hepatopancreas. When non-toxic species instead of the toxic algae were provided to mussels, a decline of toxin concentration was immediately observed in all conditions. After 14 days of depuration, the total toxin levels were decreased to low values. It can be found that the toxin mainly accumulated in the hepatopancreas on the 17th day with the PST concentration decreased to 98.5, 0.43, 226 and 27.4 μg STX eq. kg^−1^ for DT1 to DT4, while trace toxins were detected in other tissues, ranging from 0.43 to 17.5 μg STX eq. kg^−1^ under four conditions.

### 2.3. Toxin Compartmentalisation

**Figure 3:**
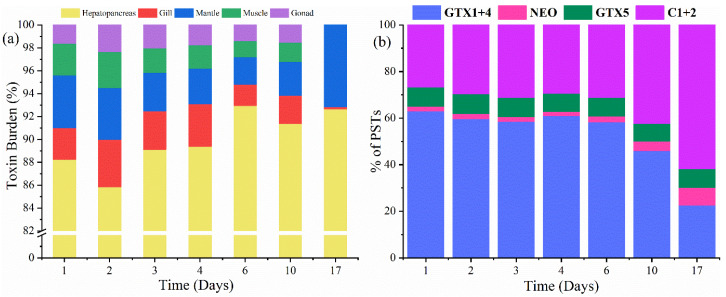
(**a**) Toxin distribution in different tissues in mussels; (**b**) profiles of PSTs (in terms of percentages) from mussels under DT3 condition.

The highest levels of PSTs were observed in DT3 condition. Hence, the toxin distributions under DT3 condition quantified within the shellfish tissues are further illustrated in Figure 3a. During all 17 days, the hepatopancreas contained most of the burdens at 86–93%. From the 1st day of the experiment to the 10th day, similar toxin burdens of 2–4% and 2–5% were observed for the gill and mantle, respectively, followed by the muscle (2–3%) and gonad (1–2%). On the 17th day, most of the toxin burdens shifted towards the mantle (7.8%), followed by little burden (0.2%) in gill and no toxin concentration in muscle or gonad (Table 2). The profile of PSTs mainly found in mussels is consistent with the content of toxic algae (>1% of total toxin, Table S1), namely GTX1+4, C1+2, GTX5 and NEO. During days 1–6, the percentage of the PSTs profile was stable with GTX1+4 and C1+2, accounting for 59–63% and 27–31% of total PSTs, respectively (Figure 3b). In comparison, C1+2 was the dominant analogue on the 17th day, constituting approximately 62% of the toxin profile observed. The GTX1+4 obviously decreased to 23%. NEO (2–7%) was a minor contributor to the toxin profile of the contaminated feed mussels. Slightly higher proportions of GTX5 (8%) were also observed.

### 2.4. PSTs Accumulation and Elimination under High Temperature and Abundance Conditions

**Figure 4:**
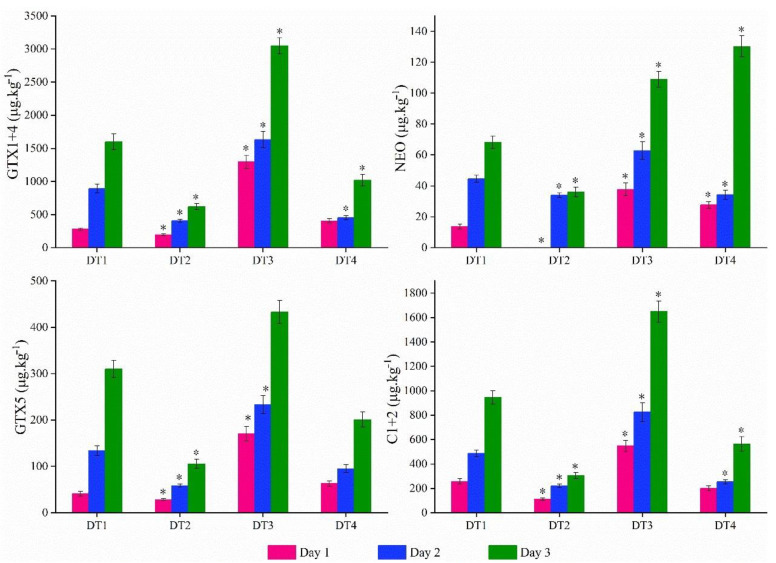
PSTs analogues concentration (µg kg^−1^, mean ± SD, GTX1+4, NEO, GTX5 and C1+2) determined in hepatopancreas at day 1 (pink bars), day 2 (blue bars) and day 3 (green bars) under four environmental conditions. Asterisks denote significant differences (*p* < 0.05) from basic conditions (vs. DT1).

Considering PSTs mainly accumulated in the hepatopancreas, the distribution of PSTs in the hepatopancreas of mussels are determined (Figure 4). After a 3-day uptake period, concentrations of PSTs congener under DT3 condition were significantly higher than any other treatment. The concentrations of GTX1+4, GTX5 and C1+2 were determined in the rank of DT3 > DT1 > DT4 > DT2. However, the content of NEO in DT4 showed slightly higher levels than that of DT3, followed by the concentration values obtained in DT1 and DT2. In addition, concentrations of all the PSTs reached the lowest values in DT2. All the above observations indicated that the concentration of PSTs were closely related to the SST and TAA.

**Figure 5:**
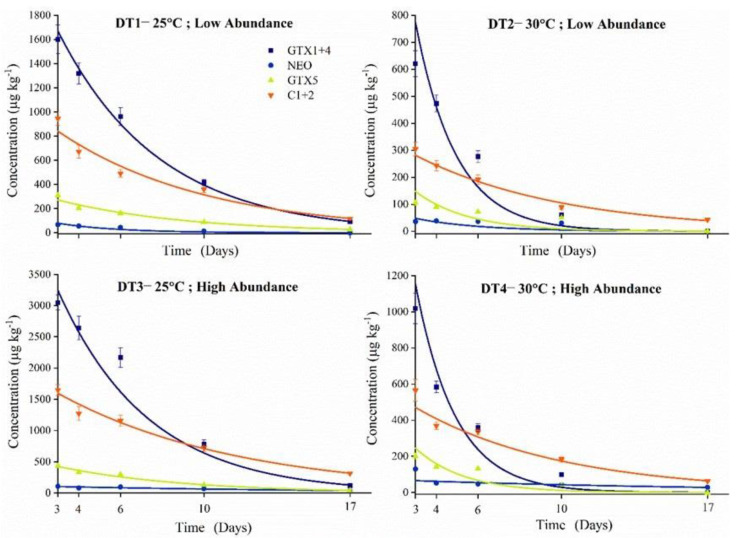
PSTs (GTX1+4, NEO, GTX5 and C1+2) elimination in hepatopancreas under four conditions. The colored lines mean the output of the simulation model.

After three days’ uptake period, the non-toxic diet for 14 days led to a significant decline of toxin concentrations during the depuration period. Nevertheless, toxins were not totally metabolized until the experiment finished. As shown in Figure 5, all PSTs analogues were consistent with an exponential decay model, with the corresponding data listed in Table 3. The highest total elimination rate was obtained in DT2, and the lowest one was calculated in DT3. The combination of high SST and TAA afforded a moderate total elimination rate, when compared to those of DT2 and DT3. In addition, the basic condition showed a similar total elimination rate relative to that of DT4.

Moreover, GTX1+4 was the main PSTs detected in mussels under the four conditions, as well as achieving the most rapid elimination rate except for that under the DT1 condition. The slowest elimination rates of NEO were calculated to be 0.069 d^−1^ (day^−1^) and 0.060 d^−1^ under DT3 and DT4 conditions, while the elimination rate values of 0.351 d^−1^ and 0.322 d^−1^ were determined under DT1 and DT2 conditions, which were obviously higher than those in DT3 and DT4. This result indicated that NEO was difficult to be removed under high toxic algal abundance condition. The elimination rates of C1+2 were calculated to be 0.140 d^−1^, 0.141 d^−1^, 0.116 d^−1^ and 0.142 d^−1^ under DT1 to DT4 conditions, respectively. It can be found that there were no significant differences between four treatments in the elimination rates of C1+2, meaning that the SST and TAA had a slight effect on the elimination for C1+2. Moreover, the elimination rates of GTX5 in DT2 and DT4 were registered to be 0.393 d^−1^ and 0.432 d^−1^, which were much higher than those of DT1 (0.206 d^−1^) and DT3 (0.232 d^−1^). These results demonstrated that the elimination rates for GTX5 were predominantly affected by the SST.

## 3. Discussion

Marine environmental change is a complicated phenomenon, resulting from temperature, pH, salinity, oxygen levels, even frequency and intensity of HABs, which has a great impact on marine organisms. Thus, it is important to investigate the coactions of these variables, which may result in multifactorial responses [52,53,54,55,56]. While studies about the effect of individual environmental change stressors on aquatic organisms have been widely reported, such as temperature [39], acidification [57] and salinity [58], research assessing their combined impacts have rarely been studied. Herein, the environmental changes regarding seawater surface temperature (SST) and toxic algal abundance (TAA) were investigated to better understand the separated or combined effect on mussels during the uptake and elimination period.

In this study, mussels were fed on PSTs-producing dinoflagellate *A. catenella*, resulting in a rapid and large accumulation of PSTs (Figure 2), which exceeded the EU regulatory limit of 800 µg STX eq. kg^−1^ [59] when maintained under basic conditions (DT1), high TAA conditions (DT3,) and combined conditions (DT4). In contrast, mussels acclimated solely to high SST conditions (DT2) showed the PSTs of 741 ± 51 μg STX eq. kg^−1^, which was below but close to the safety limit. These results demonstrated that the increase of environmental temperature can significantly reduce the accumulation of toxin. In the respect, the PSTs levels in *S. glomerata*, diploid *C. gigas* [39], and *M. galloprovincialis* [38] were obviously decreased under high temperature conditions during the uptake period.

In general, HABs expand geographically, thus producing various toxic algae abundance in sea areas, which result in different biotoxin concentrations in marine organisms. Therefore, the TAA was investigated to understand the role on PSTs accumulation, and mussels were cultured in a high TAA scenario with (DT4) and without high SST (DT3). As expected, significantly increased toxicity in mussels was observed under DT3 condition, with the highest 3548 ± 299 μg STX eq. kg^−1^ PSTs toxicity level (Figure 2). Xie [17] investigated PSTs accumulation in the oyster *Ostrea rivularis* Gould (ORG), which was fed with three abundances of toxic algae *Alexandrium minutum*. After the feeding experiment, the toxic levels of ORG showed high levels of 28.86, 31.17 and 38.98 MU/g in 4.0 × 10^3^, 8.0 × 10^3^ and 1.2 × 10^4^ cells/mL, respectively. These observations can be ascribed to the higher intake of mussels, accompanied by the increased TAA in the environment. With the contradictory trends of high SST and TAA, mussels showed a PSTs level of 1363 ± 108 μg STX eq. kg^−1^ under combined conditions (DT4), which was much lower than that of DT1. This result indicated that the toxicity levels in mussels were mainly dominated by the temperature under the combined condition, which can be rationalized by the high depuration rates in the toxin accumulation period at high SST.

Due to the different function of depuration for each tissue, the toxins in the shellfish are not equally distributed. In this study, the hepatopancreas contained the highest toxin burden, followed by the mantle, gill, muscle and lastly by the gonad (Figure 1 and Figure 3a). Obvious changes of toxin profiles were observed on the last day, wherein the mantle contained 7.8% toxin levels and the gill had little burden at 0.2%. Meanwhile, no toxin was detected in the muscle or gonad (Figure 3a, Table 2). These observations are consistent with those reported in the literature showing that PSTs have a tendency to accumulate in the hepatopancreas for mussels with the highest burden [28,60,61]. Moreover, it is reported that PSTs can quickly accumulate in the hepatopancreas of *Jasus edwardsii* if they are exposed to high toxicity, exceeding the maximum levels for bivalve molluscs [27]. We may interpret this to mean that the hepatopancreas is the main organ where toxins are exposed, absorbed, ingested, and metabolized. Moreover, the gill, mantle, muscle and gonad were contaminated by toxins in mussels but are found to have the lowest toxicity. The PSTs profiles of contaminated mussels revealed that the principal components were GTX1+4 and C1+2, which together made up almost 90% of the total. These profiles were comparable with those reported for *A. catenella* strains [62], in which the presence of *A. catenella* in mussels was determined and a similar PSTs profile was found. It is noteworthy that the dominant analogue detected in the algal profiles was GTX1+4. Conversely, C1+2 was found to be higher at the end of the experiments. This change in potency is advantageous to the mussels, as it results in a reduced total toxicity relative to molar concentration of toxin present. Different processes for toxin biotransformation include reduction, epimerization, oxidation, and desulfation [63,64,65]. The biotransformation potentially observed in this study resulted in the conversion to a less potent (C1+2) from a more potent (GTX1+4) form, which may be due to the epimerization process [66], and/or with the help of enzymes [67]. However, our study was not designed to discuss biotransformations. Thus, we cannot confirm the mechanism that drive the change in toxin proportions.

Considering the change of SST and TAA brought about by the various PSTs toxicity levels, the concentrations of PSTs analogues were further determined during accumulation and elimination period. The carbamate analogues (GTX1+4 and NEO) were the most abundant toxin congeners detected in all conditions in mussels, which coincided with the content of the administered algae (Table S1). The toxicity level of GTX1+4 was nearly twice as high under DT3 condition than that of DT1 on day 3 (Figure 4). However, the concentrations of GTX1+4 were obviously lower under DT2 conditions and the combined condition in DT4, which indicated that high TAA tended to cause GTX1+4 accumulation in the mussels while high SST brought a toxicity depression. A similar tendency was observed in N-sulfocarbamoyl toxins C1+2 and GTX5. Additionally, GTX1+4 was the most potent among the PST derivatives (Table S2), significantly impacting PSTs toxicity in mussels. From our observation, the increase of environmental temperature was the dominated element to decrease toxicity levels in mussels, which can be attributed to their enhanced abilities in clearance, ingestion and metabolism [68,69,70]. On the other hand, the trend of NEO toxicity levels was greatly different from GTX1+4 under four conditions. The highest value was observed under the combined conditions of DT4, followed by that of DT3 condition, which illustrated that high TAA-acclimated mussels were contaminated with much more NEO toxin.

The effects of environmental change also strikingly differed between the PST analogues during the depuration period. The concentrations of GTX1+4, C1+2, NEO and GTX5 detected in mussels fit well with the dynamic model (Figure 5). The elimination rates of GTX1+4 were much higher under DT2 and DT4 conditions, which demonstrated that increasing SST may promote the elimination, further reducing the toxin level in mussels. Similar trends were also observed for GTX5. Although the highest concentrations of C1+2 were obtained under four conditions followed by GTX1+4, low elimination rates in mussels were calculated for C1+2 under all conditions, resulting in the mussels being contaminated for longer periods. However, the variations of C1+2 concentrations showed less influence on total PSTs toxicity due to the low potency. The reverse trend was described by Braga [38], reporting a decrease in both accumulation and elimination of C1+2 warming-acclimated mussels fed with toxic *G. catenatum*. The lowest elimination rate was obtained for NEO in high TAA-acclimated mussels (DT3, 0.060 d^−1^) and under combined conditions (DT4, 0.069 d^−1^), which were dramatically lower than those under the low TAA conditions such as DT1 and DT2 (0.351 d^−1^ and 0.322 d^−1^). Under the circumstances of high TAA conditions, some digestive enzymes may have higher potencies, accompanied by a reduction in glutathione consumption, which plays an important role in biotransformation and elimination of PSTs in vivo [71,72,73,74]. In the southern rock lobster *Jasus edwardsii*, the uptake and depuration rates of PSTs changed among analogues, resulting in a reduction of the highly potent analogues in lobster tissues [27]. Particularly, the elimination rate of GTX1+4 was obviously more rapid than those of most other analogues.

The increase in environmental temperature caused a significant reduction in PSTs of mussels, whereas high toxic algal abundance led to a dramatic enhancement in toxicity. As a result, the combined factors illustrated the lower toxicity of these mussels compared to those subjected to the basic condition, which demonstrated that the increasing temperature within a reasonable range was a good way to reduce the PSTs of mussels.

## 4. Conclusions

The effects of environmental changes, high seawater surface temperature (SST), high toxic algal abundance (TAA) and the combination of these conditions were investigated using the PSTs accumulation and elimination in mussels. It was observed that high SST and TAA caused the contradictory variation trends in mussels, *Mytilus coruscus*. The enhancement of SST resulted in the reduction of toxicity levels and rapid elimination rates, while high TAA tended to cause more biotoxin accumulation and prolonged PSTs contamination. As a result, the combined effects of high SST and TAA promoted lower PSTs than that under the basic condition, indicating that the toxicity was mainly dominated by the high SST due to the rapid depuration rate. Considering the toxin distribution, toxin burdens in the hepatopancreas were assessed to be the highest level after 3 days in mussels. Moreover, it can be also found that the elimination rates of each PSTs analogue strikingly differed under the variety of conditions. As a result, this study provided an approach for shellfisheries affected by *A. catenella* blooms, confirming the importance of both environmental temperature and toxic algal abundance monitoring for managing the risks from PSTs in mussels.

## 5. Materials and Methods

### 5.1. Reagents and Chemicals

Certified reference materials of STX, NEO, C1/2, GTX1/4, GTX2/3, GTX5, dcSTX, dcNEO and dcGTX2/3 were ordered from the National Research Council Canada (Halifax, NS, Canada). All reagents and chemicals used in this study were analytical or liquid chromatography grade. Methanol and acetonitrile were purchased from Merck Ltd. (Whitehouse Station, NJ, USA); acetic acid, formic acid and ammonium formate from Sigma-Aldrich. De-ionized water (18.2 MΩ cm quality or better) was obtained from a Milli-Q water purification system (Millipore Ltd., Bedford, MA, USA).

### 5.2. Culture of A. catenella

The PSTs-producing strain of *A. catenella* GY-H31, purchased from the algae culture collection at Shanghai Guangyu Biological Technology Co., Ltd. (Shanghai, China), was isolated from a bloom in the estuary of the Changjiang River in September 2018. Cells were cultured in sterile-filtered (0.45 μm membrane, Jinjing Ltd., China) seawater before enrichment with f/2-Si medium [75]. Temperature and photon flux density (Asensetek, ALP-01, Shanghai HESON Instrument Technology Co., LTD, Shanghai, China) were set at 25 °C and 60–100 µmol photon m^−2^ s^−1^, respectively, with a 14 h light: 10 h dark cycle. Seawater and nutrient solutions were filtered and autoclaved to minimize contamination. Cells were harvested when cultures presented a density of approximately 2.5 × 10^6^ cells per liter, concentrated using 10 µm mesh sieve. Toxins were determined in the algae cell culture as described below (Section 5.5). Cells of microalgae were collected at the stationary growth phase and used to feed experimental mussels.

### 5.3. Mussel Collection and Acclimation

Two hundred uncontaminated mussels, *Mytilus coruscus* (80 ± 8 mm), were harvested in Shengsi Archipelago, China, in May 2020. Mussels were cleaned from macro-algae, barnacles or any other epibiont in four 250 L tanks of filtered seawater with continuous aeration. The mussels were acclimatized for 14 days at 24 ± 2 °C and 30 ± 2 °C, half and half, being fed a non-toxic *Chlorella vulgaris* diet (Huayi Biological Technology Co., Ltd., Suzhou, China). The temperature was automatically adjusted whenever needed. Water temperature was cooled through an automatic seawater refrigeration system (±0.1 °C; Frimar, Fernando Ribeiro Lda, Portugal) or heated by submerged digital heaters (200 W, V2Therm, TMC Iberia, Lisbon, Portugal). Eight mussels were randomly selected to assess background toxin profiles before the feeding experiment.

### 5.4. Mussels Exposure to Toxic Dinoflagellates

During acclimation, mussels were fed with 100,000 cells per day per animal of the non-toxic *Chlorella vulgaris* diet. The mussels were then fed for 3 days with toxic dinoflagellate from a *A. catenella* culture under the conditions indicated in a specific treatment: DT1–basic condition (25 °C; 5.6 × 10^6^ cells L^−1^ *A. catenella*), DT2–high SST (30 °C; 5.6 × 10^6^ cells L^−1^ *A. catenella*), DT3–high toxic algal abundance (TAA) (25 °C; 1.0 × 10^7^ cells L^−1^ *A. catenella*) and DT4–high SST and TAA (30 °C; 1.0 × 10^7^ cells L^−1^ *A. catenella*) (Figure 1). After 3 days, mussels were fed again with 100,000 cells per day per animal of *Chlorella vulgaris* for 14 days in order to evaluate the elimination of toxins accumulated during the exposure period. Six mussels exposed to *A. catenella* were collected in triplicate for toxin analysis on days 1, 2 and 3, corresponding to the uptake period, and on days 4, 6, 10 and 17, corresponding to the elimination period.

### 5.5. Toxin Extraction

Extraction of toxins from *A. catenellae* cell cultures followed the methodology described by Silva [76]. Briefly, an aliquot of *A. catenella* cell culture (500 mL) was filtered onto 47 mm Whatman GF/C with a nominal pore size of 1.2 µm under low vacuum. Toxins were extracted in 5 mL of 1% acetic acid solution [77] and sonicated for 10 min using a sonication probe (Branson, Emerson, Saint Louis, MO, USA). The extract was then centrifuged (10,000× *g*) for 10 min at 4 °C, and 1 mL of the supernatant was decanted into a vial for the determination of PSTs.

For experimental sampling, the mussel was dissected into five parts, collecting the hepatopancreas, gill, gonad, mantle and muscle, all of which were homogenised (VELP OV5) and frozen at −80 °C. Then, 5 g of shellfish tissues were extracted with 5 mL 1% acetic acid solution. Then, the tissue samples were vortex mixed then placed in a boiling water bath for ten minutes. After that, the sample was cooled down to room temperature, centrifuged (10,000× *g*) for 10 min at 4 °C, and then 1 mL aliquot was transferred into a 1.5 mL polypropylene tube, followed by the addition of 5 µL of ammonium hydroxide (NH_4_OH; 25% ammonia) before clean-up. The supernatant was cleaned by solid-phase extraction (SPE) with an octadecyl bonded phase silica (Supelclean LC-18 SPE cartridge, 3 mL, Supelco, Bellefonte, PA, USA). Briefly, the SPE cartridge was conditioned with 3 mL of acetonitrile and 3 mL of 1% acetic acid in 20% acetonitrile, followed by 3 mL of 0.1% aqua ammonia. Afterwards, 0.5 mL of extracts were loaded onto the conditioned cartridges and then washed with 700 µL of MilliQ water. PSTs were eluted with 1 mL of 0.25% formic acid in 75% acetonitrile. All sub-samples were taken and filtered through 0.22 μm membrane prior to analysis.

### 5.6. LC–MS/MS Analysis

For common analogues of PSTs, selective reaction monitoring (SRM) was used. A Shimadzu DGU-20A5R HPLC was coupled with a Sciex Qtrap 5500 tandem quadrupole mass spectrometer (Danaher Corporation, Washington, DC, USA) with an electrospray ionization interface. The chromatographic separation was performed on a TSK-gel Amide-80^®^ HILIC column (150 × 2 mm i.d., 3 μm, Tosoh Bioscience LLC, Montgomeryville, PA, USA) using a flow rate of 1.2 mL min^−1^ at 40 °C. A binary mobile phase of water (solvent A) and 95% acetonitrile (solvent B), each of them containing 2 mM ammonium formate and 50 mM formic acid. The gradient ran from 90 to 80% B over 3.6 min, decreasing to 60% B over an additional 2.4 min, held for 1.5 min at 60% B, increasing to 90% B over an additional 1 min, held for 1.5 min at 90% B before re-equilibration for the next run. High resolution mass spectrometry conditions included a spray voltage of 5.0 kV and −4.5 KV for positive and negative ions, respectively, a CUR pressure of 20 psi, GS1 and GS2 pressure of 30 psi, ion source temperature of 550 °C, and CAD of medium [63,78]. Common analogues were scanned using the SRM transitions shown in Table 4 and the total ion chromatogram and mass spectrum are shown in the Supplementary Materials (Figures S2–S12). Relative toxin STX equivalency factors (Table S2) are from FAO/IOC/WHO (2004) background document on biotoxins in bivalve mollusks [79,80].

### 5.7. Statistical Analysis

Analysis of covariance (ANCOVA) was performed to examine toxin concentrations in four conditions over time, with the weight of mussels as a covariate.

For the empirical kinetics of PSTs elimination, a one-compartment model was used to describe elimination kinetics using a single component first-order kinetic model:

(1) $$C_{m}=C_{m0}e^{-k_{el}t}$$

where *C_m_* is the toxin concentration in mussels and *k_el_* denotes the elimination rate. The toxin concentration decreases according to an exponential decay, with the steepness of the decay being determined by the elimination rate (*k*) and the size of the curve depending on the initial concentration of the toxin (C*_m_*_0_) at the beginning of the elimination period, when the mussels’ diet was changed from *A. catenella* to non-toxic algae. All statistical analyses were performed using the software SPSS version 22.0 (SPSS Inc., Chicago, IL, USA). The data were analyzed and compared using a Student’s *t*-test. Differences between means were considered significant at the *p* < 0.05 level.

## Figures and Tables

**Table 1:** Toxin data of *A. catenella* fed to mussels.

Toxins	Toxin Concentration (pg/cell)	% of Total Toxin
C1+2	6.69	38.9
GTX1+4	8.86	51.6
GTX2+3	0.053	0.3
GTX5	1.15	6.7
dcGTX2+3	0.055	0.4
STX	0.018	0.1
NEO	0.319	1.9
dcSTX	0.019	0.1

**Table 2:** The distribution of analogues in different tissues and PST profiles (percent molar).

Days	% of Toxin Distribution					% of the PSTs			
	Hepatopancreas	Gill	Mantle	Muscle	Gonad	GTX1+4	NEO	GTX5	C1+2
1	88	3	4	3	2	63	2	8	27
2	86	4	5	3	2	60	2	8	30
3	90	3	3	2	2	59	2	8	31
4	89	4	3	2	2	61	2	8	29
6	93	2	2	2	1	59	2	8	31
10	91	2	3	2	2	46	4	8	42
17	92	0.2	7.8	-	-	23	7	8	62

**Table 3:** Elimination rates (*k*_el_, d^−1^) and coefficient of determination R^2^ under four conditions.

Treatment	Toxin	Elimination Rate		R^2^
DT1 25 °C and Low abundance	GTX1+4	0.206	(±0.004)	0.9967
	NEO	0.351	(±0.064)	0.8762
	GTX5	0.163	(±0.007)	0.9865
	C1+2	0.140	(±0.012)	0.9765
DT2 30 °C and Low abundance	GTX1+4	0.518	(±0.082)	0.8264
	NEO	0.322	(±0.116)	0.6666
	GTX5	0.393	(±0.099)	0.7592
	C1+2	0.141	(±0.011)	0.9766
DT3 25 °C and High abundance	GTX1+4	0.232	(±0.014)	0.9791
	NEO	0.069	(±0.011)	0.9377
	GTX5	0.167	(±0.006)	0.9912
	C1+2	0.116	(±0.004)	0.9948
DT4 30 °C and High abundance	GTX1+4	0.526	(±0.095)	0.7638
	NEO	0.060	(±0.026)	0.6236
	GTX5	0.432	(±0.098)	0.8189
	C1+2	0.142	(±0.009)	0.9828

**Table 4:** Acquisition parameters of SRM mode scanning for paralytic shellfish toxins.

ESI Mode	Toxin	Precursor Ion (*m*/*z*)	Product Ion (*m*/*z*)	Fragmentor (v)	Collision Energy (v)
ESI^−^	GTX2,3	394.0	333.1	80	−22
			351.1		−16
	GTX1,4	410.1	367.1	80	−15
			349.1		−22
	dcGTX2,3	351.1	333.1	100	−17
			164.0		−30
	C1/2	474.1	351.1	90	−25
			122.0		−30
	GTX5	378.1	122.1	100	−22
			360.1		−16
ESI^+^	STX	300.2	221.0	120	35
			204.0		30
	NEO	316.1	298.2	120	34
			126.1		34
	dcSTX	257.1	239.1	120	22
			126.1		30
	dcNEO	273.1	225.2	120	35
			126.1		35

## Data Availability

The data presented in this study are available within this article and its Supplementary Materials.

## Supplementary Materials

The following are available online at https://www.mdpi.com/article/10.3390/toxins13060425/s1: Table S1—Acquisition parameters of SRM mode scanning for paralytic shellfish toxins; Figure S1—PSTs (µg STX eq. kg^−1^, mean ± SD) determined in different tissues of mussels exposed to toxic *A. catenella* under four environmental conditions.
