# Supplementary material for: Combined Effects of Temperature and Toxic Algal Abundance on Paralytic Shellfish Toxic Accumulation, Tissue Distribution and Elimination Dynamics in Mussels Mytilus coruscus
Source: Toxins (Basel). 2021 Jun 17;13(6):425. doi: 10.3390/toxins13060425 (PMC8235259; doi:10.3390/toxins13060425)
Supplement: Supplementary file 1 [file toxins-13-00425-s001.zip › toxins-1229517-supplementary from proof-doi.pdf]

Supplementary Materials: Combined Effects of Temperature and Toxic Algal Abundance on Paralytic Shellfish Toxic Accumulation, Tissue Distribution and Elimination Dynamics in Mussels *Mytilus coruscus*

Yunyu Tang, Haiyan Zhang, Yu Wang, Chengqi Fan and Xiaosheng Shen

Table S1. Acquisition parameters of SRM mode scanning for paralytic shellfish toxins.

ESI mode	Toxin	Precursor ion (m/z)	Product ion (m/z)	Fragmentor (v)	Collision energy (v)
ESI^-	GTX2,3	394.0	333.1	80	−22
			351.1		−16
	GTX1,4	410.1	367.1	80	−15
			349.1		−22
	dcGTX2,3	351.1	333.1	100	−17
			164.0		−30
	C1/2	474.1	351.1	90	−25
			122.0		−30
ESI^+	STX	300.2	221.0	120	35
			204.0		30
	NEO	316.1	298.2	120	34
			126.1		34
	GTX5	380.1	300.1	100	15
			282.1		35
	dcSTX	257.1	239.1	120	22
			126.1		30
	dcNEO	273.1	225.2	120	35
			126.1		35

Table S2. Toxicity Equivalency Factors of PSTs.

PSTs	GTX1	GTX4	GTX2	GTX3	dcGTX2	dcGTX3	GTX5	NEO	STX	dcSTX	dcNEO	C1	C2
Equivalency Factors	0.99	0.73	0.36	0.64	0.65	0.75	0.06	0.92	1	0.51	0.30	0.01	0.13

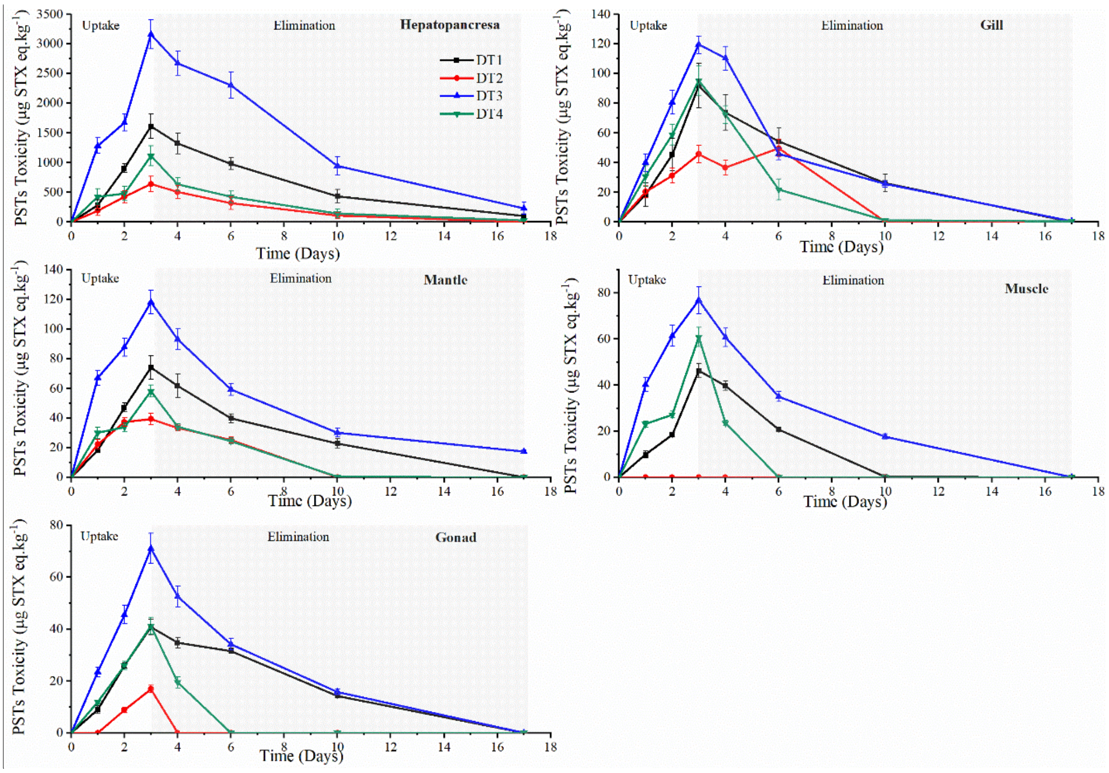

Figure S1. PSTs (μg STX eq. kg^{-1}, mean ± SD) determined in different tissues of mussels exposed to toxic *A. catenella* under four environmental conditions.

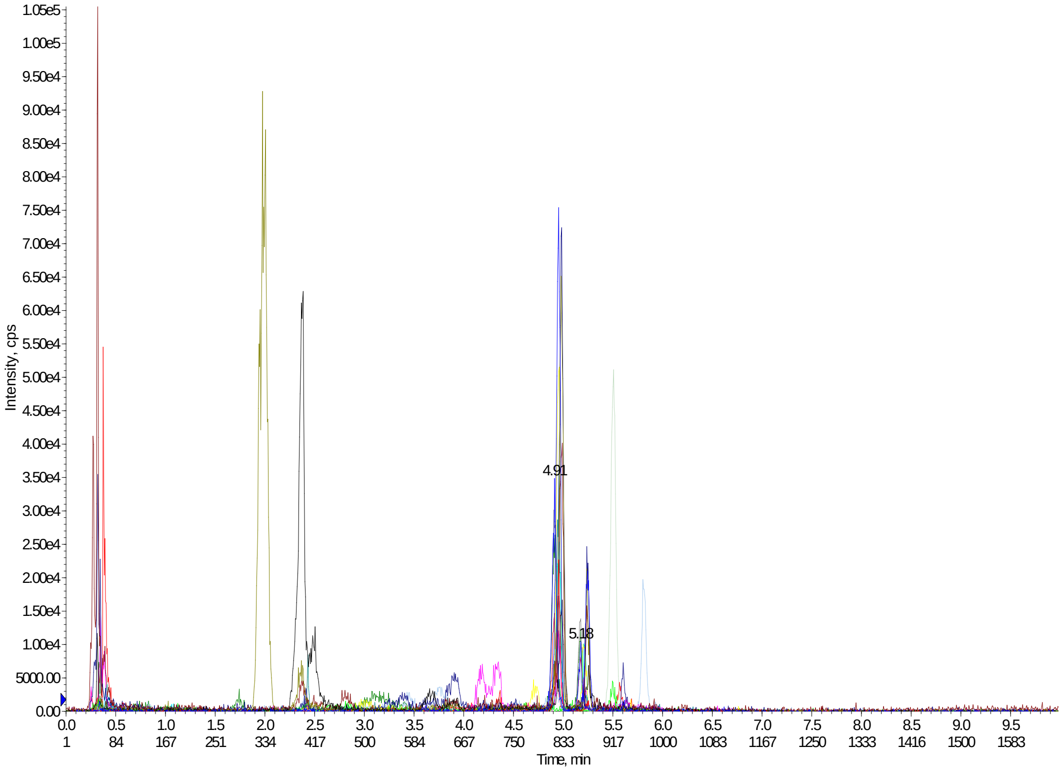

Figure S2. The total ion chromatogram of GTX2,3, GTX1,4, dcGTX2,3, C1,2 and GTX5.

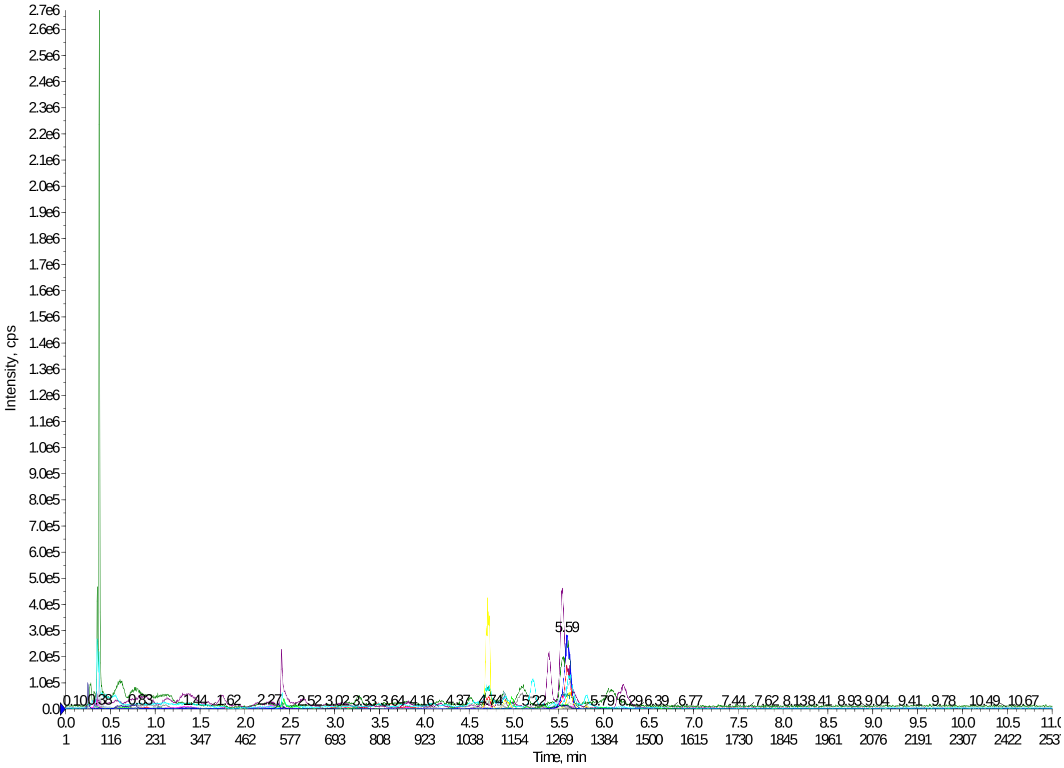

Figure S3. The total ion chromatogram of STX, NEO, dcSTX and dcNEO.

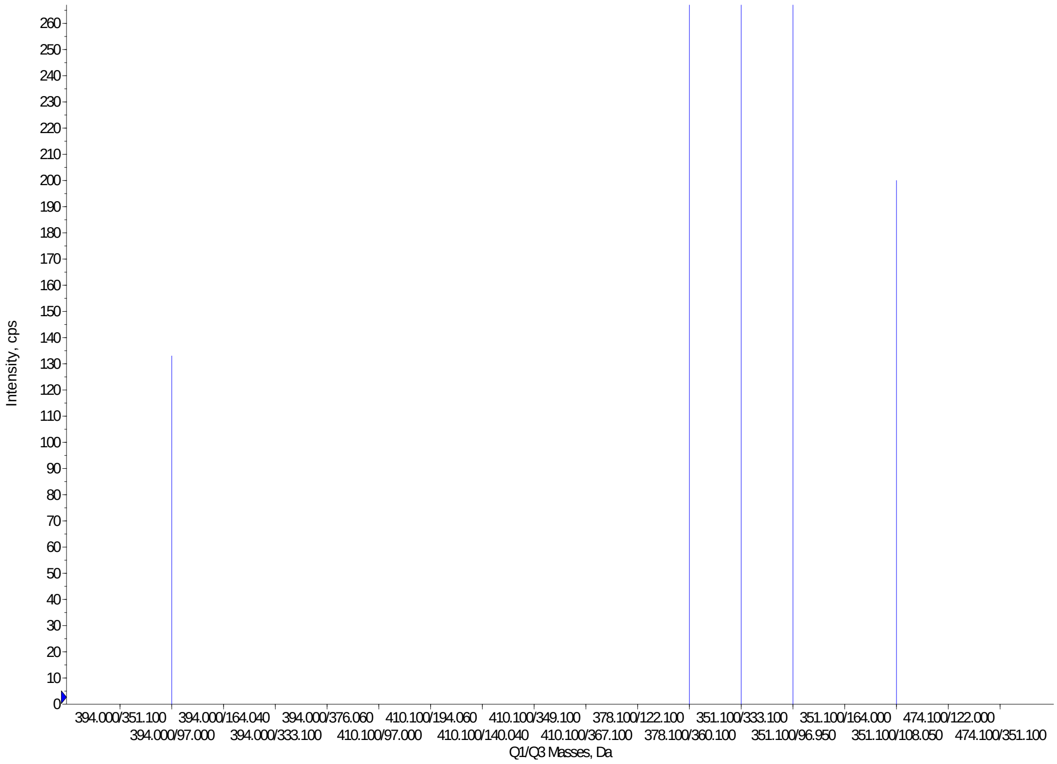

Figure S4. The mass spectrum of GTX2,3.

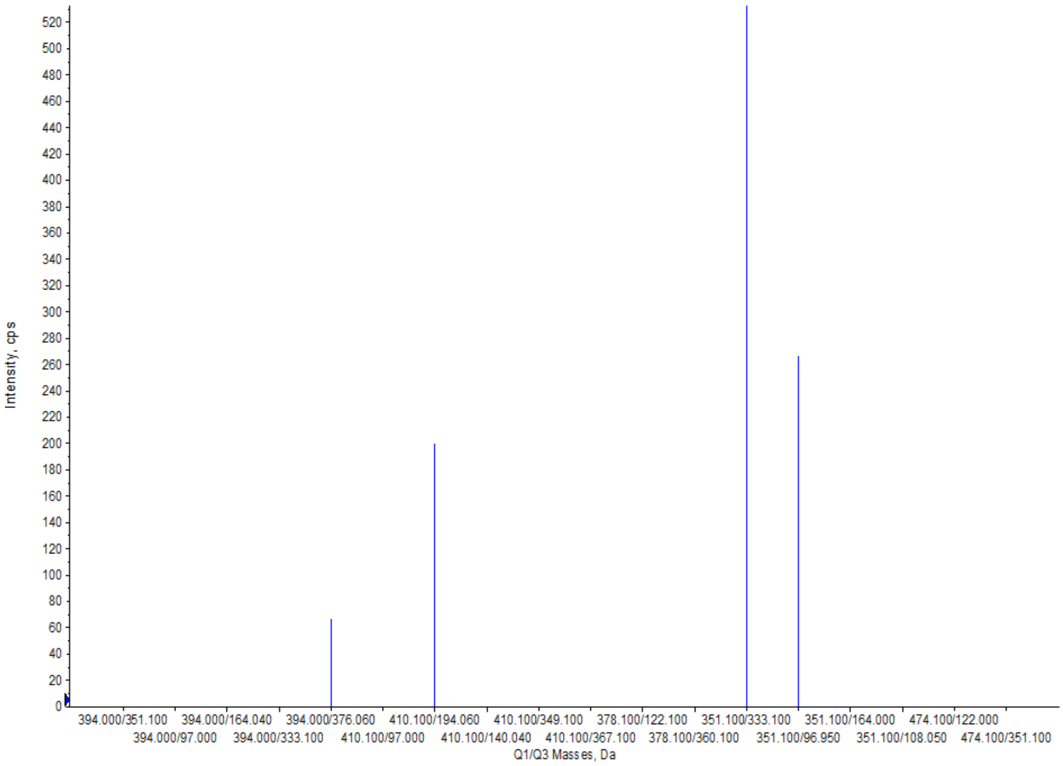

Figure S5. The mass spectrum of GTX1,4.

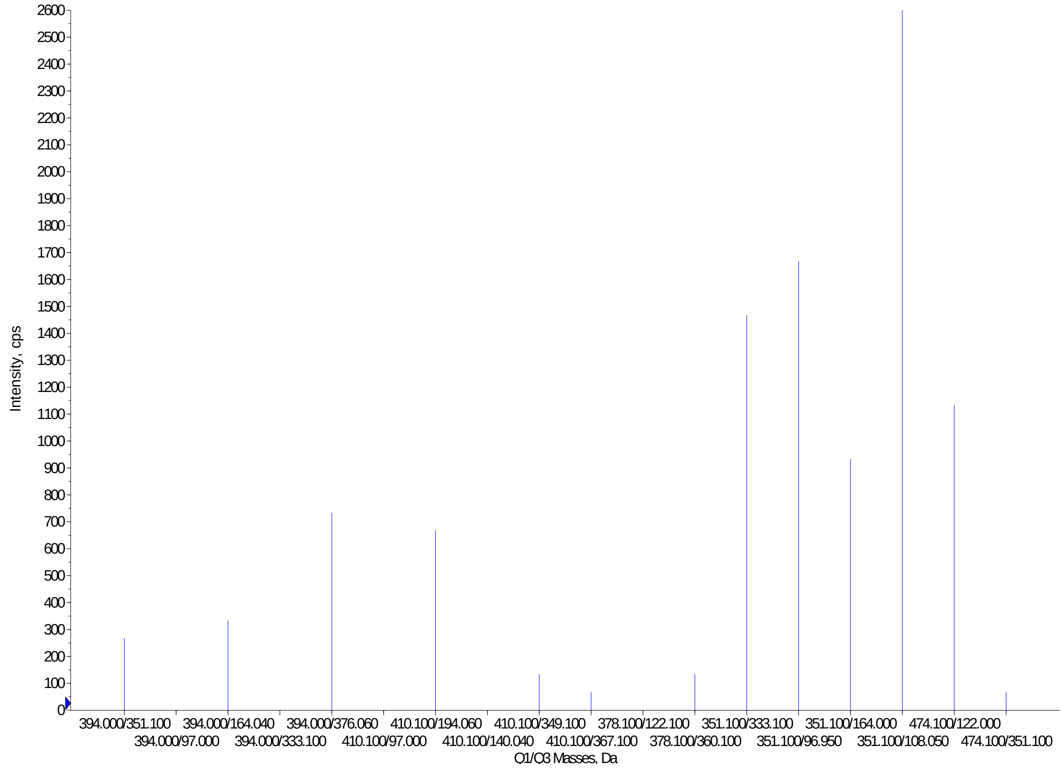

Figure S6. The mass spectrum of dcGTX2,3.

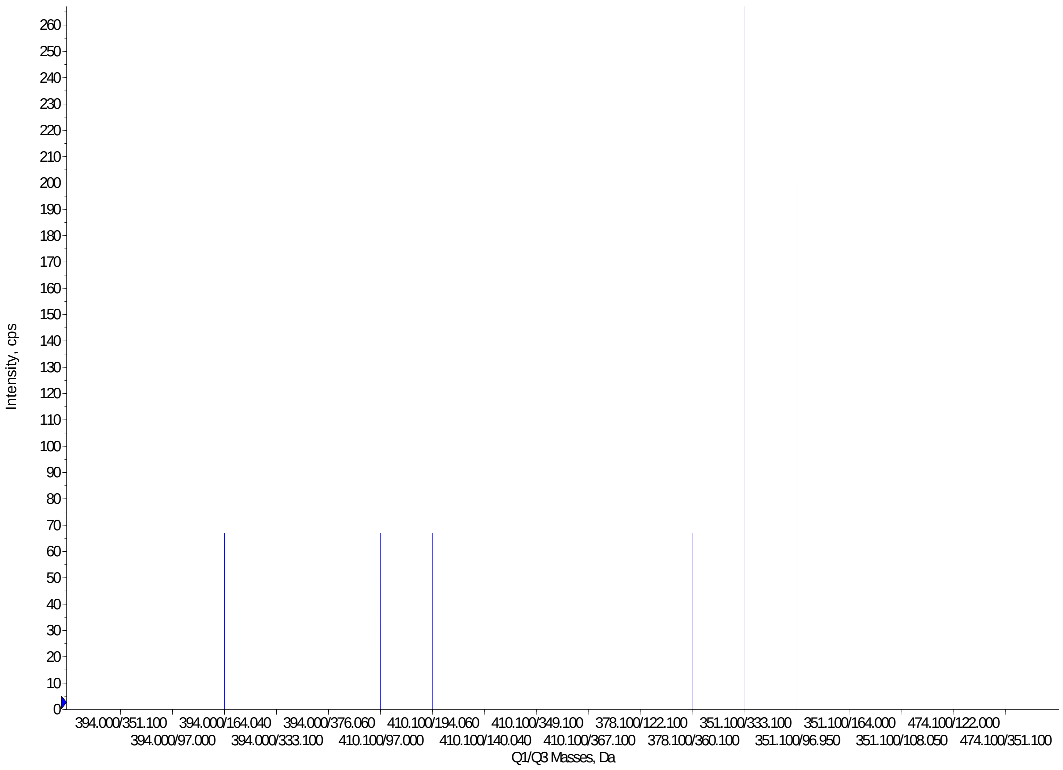

Figure S7. The mass spectrum of C1/2.

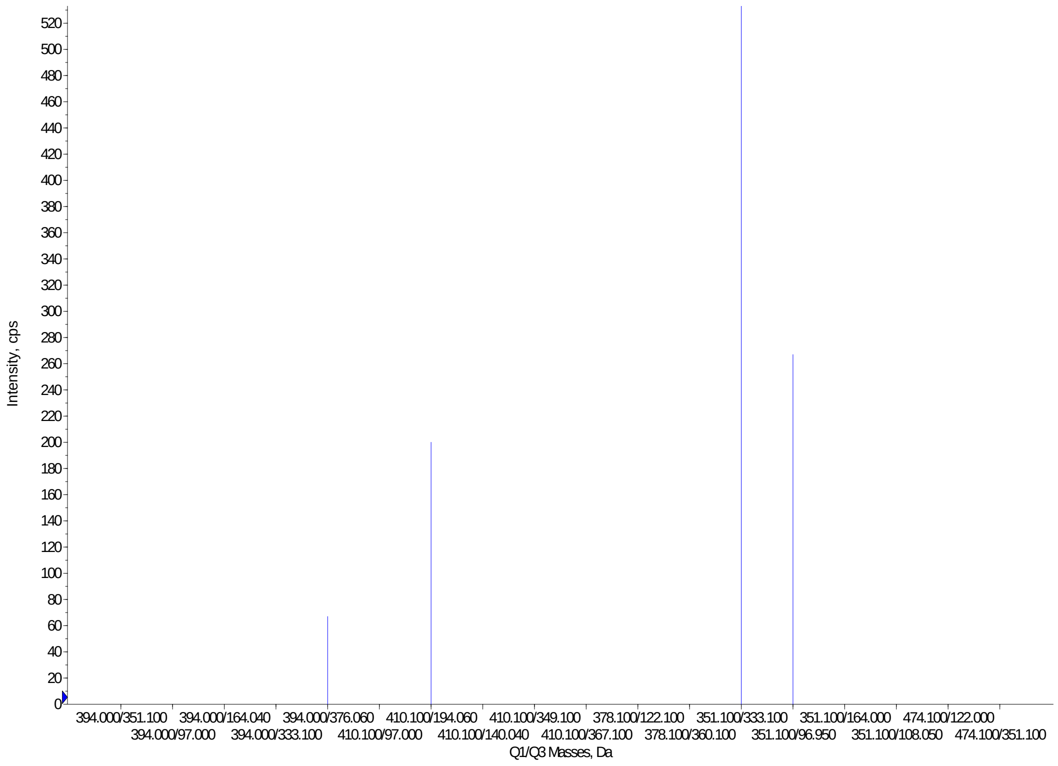

Figure S8. The mass spectrum of GTX5.

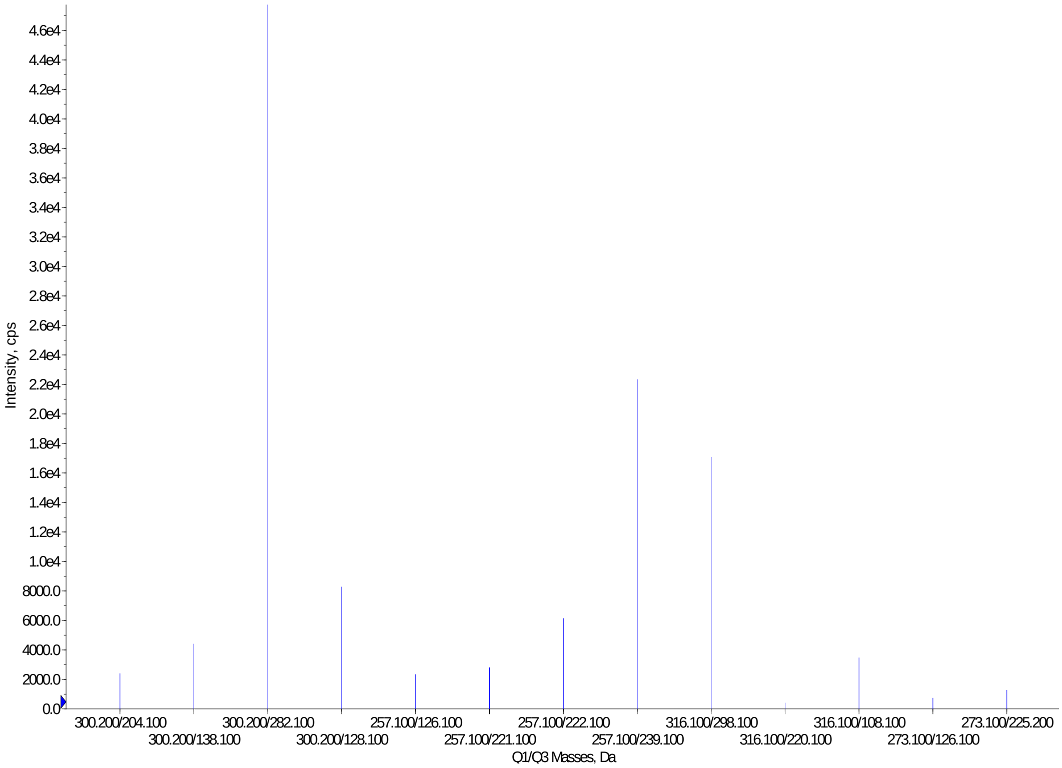

Figure S9. The mass spectrum of dcNEO.

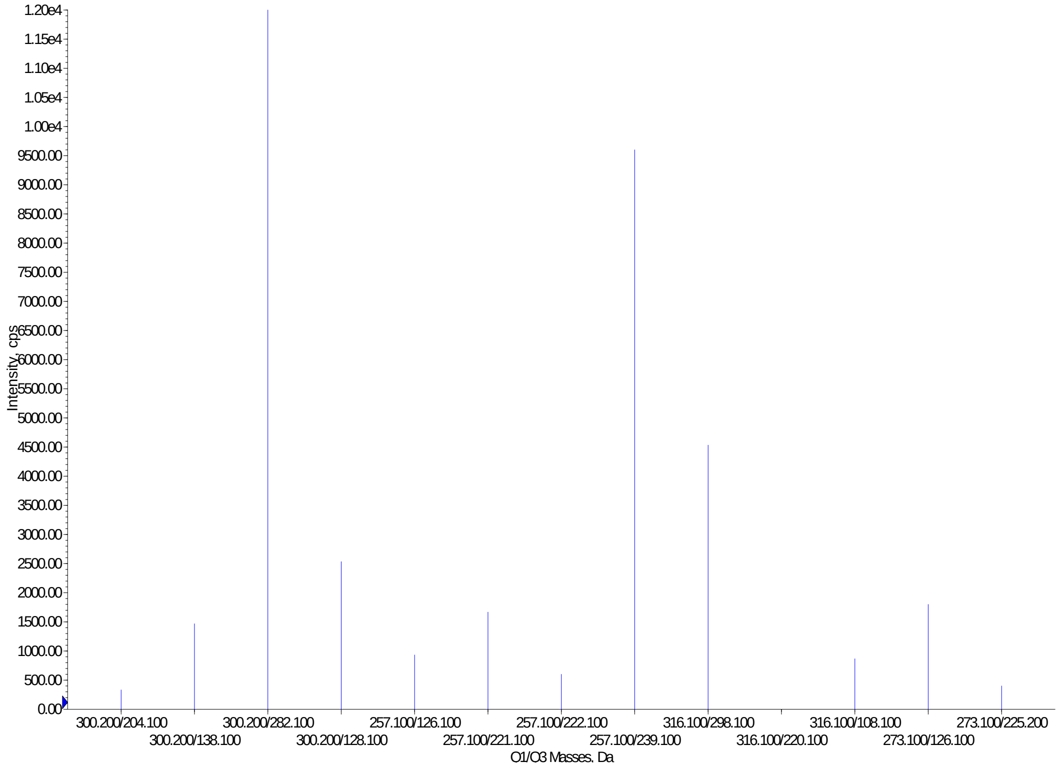

Figure S10. The mass spectrum of STX.

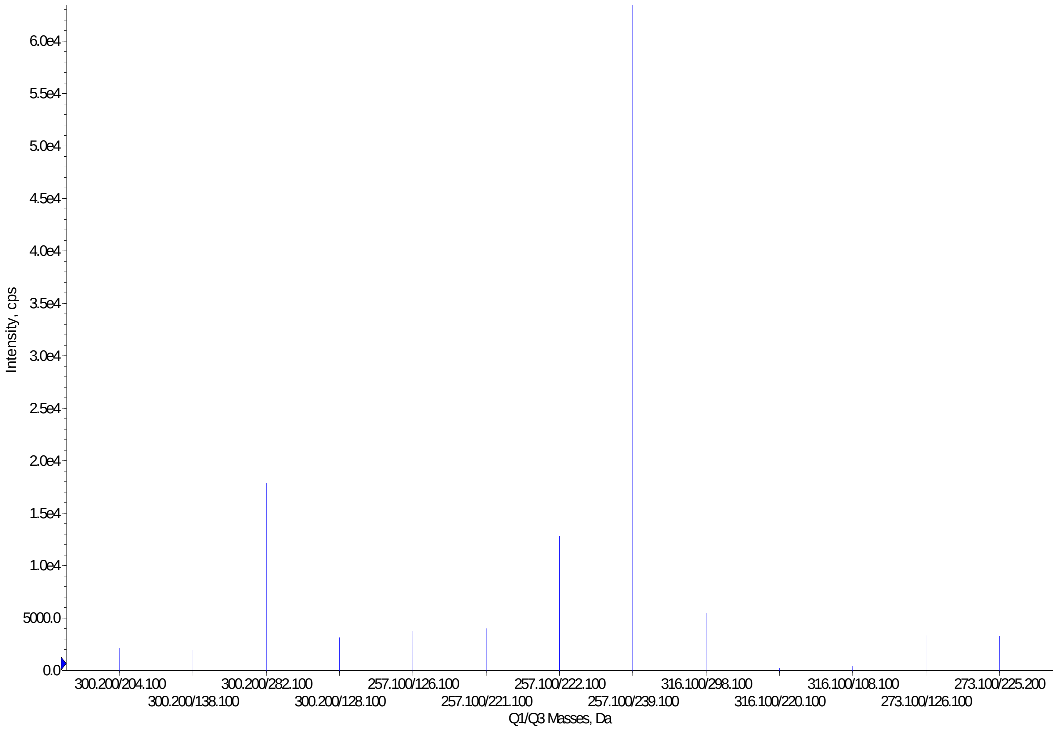

Figure S11. The mass spectrum of NEO.

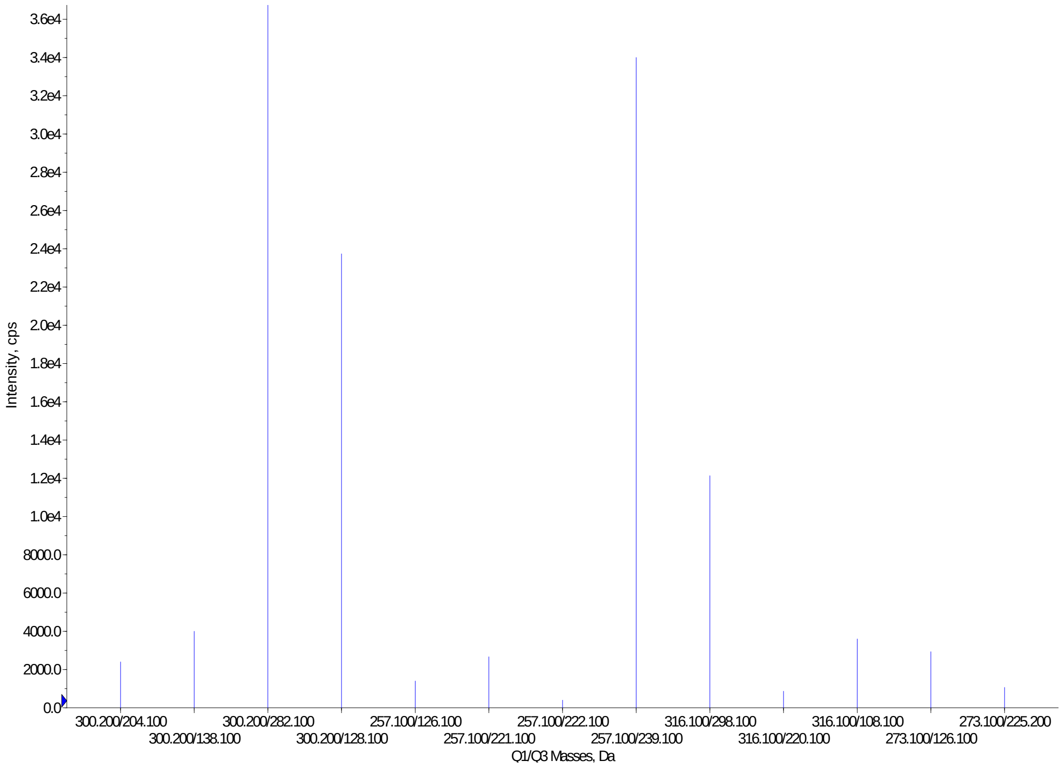

Figure S12. The mass spectrum of dcSTX.
